# The feasibility of oligogenic combined segregation and linkage analysis in CEPH pedigrees

**DOI:** 10.1186/1753-6561-1-s1-s108

**Published:** 2007-12-18

**Authors:** E Warwick Daw, Robert Yu

**Affiliations:** 1 Department of Epidemiology, University of Texas M.D. Anderson Cancer Center, Unit 1340, 1155 Pressler Street, Houston, Texas 77030, USA; 2 Program in Human and Molecular Genetics, University of Texas Graduate School of Biomedical Sciences, 1155 Pressler Street, Houston, Texas 77030, USA; 3 Program in Biomathematics and Biostatistics, University of Texas Graduate School of Biomedical Sciences, 1155 Pressler Street, Houston, Texas 77030, USA; 4 Current affiliation: Division of Statistical Genomics, Washington University School of Medicine, 4444 Forest Park Blvd, Campus Box 8506, St. Louis, MO 63108, USA

## Abstract

The CEPH samples are an invaluable resource for mapping genes that contribute to traits that can be measured in cell lines. With the many markers that have already been genotyped for the Centre d'Etude du Polymorphisme Humain (CEPH) pedigrees and are readily available, one need only obtain phenotypes to conduct a linkage analysis. For Genetic Analysis Workshop 15 (GAW15), over 3000 expression levels of genes in lymphoblastoid cells in 14 of the CEPH pedigrees were provided. For this study, eight of these expression levels were selected to obtain a spectrum of heritabilities, three were selected based on linkage results with traditional LOD scores >3, and one trait was selected at random. A Bayesian Monte Carlo Markov chain oligogenic segregation and linkage analysis was conducted on each of these 12 traits using the genome-wide single-nucleotide polymorphism linkage markers provided for GAW15. Our goal was to assess the ability of these methods to map genes in the CEPH pedigrees. Surprisingly, positive linkage signals were found for all 12 traits, even those with a very small traditionally calculated heritability. However, the portion of the variance attributed to genetic sources by the oligogenic segregation analysis differed substantially in some cases from the traditional heritability. It appears that genetic variance estimated from oligogenic segregation analysis may be a better indicator of whether genes can be mapped for complex traits than traditional heritability.

## Background

The Centre d'Etude du Polymorphisme Humain (CEPH) reference families were originally collected as a resource for constructing linkage maps. Cell lines from these families are available to the research community and have been used in many studies. As a result, many genetic markers have been typed and are available. This resource has the potential to be used to map genes for any phenotype that can be measured in cell lines.

For Genetic Analysis Workshop 15 (GAW15), gene expression levels were available for thousands of genes measured in 14 CEPH pedigrees (12 of size 14, 2 of size 13, 194 individuals total). These levels were obtained via the Affymetrix Human Focus Arrays and selected as described in Morley et al. [1]. In addition, a linkage screen of single-nucleotide polymorphism (SNP) markers was provided to participants. To characterize the ability of Monte Carlo Markov Chain (MCMC) oligogenic segregation and linkage analysis to map genes in the CEPH families, 12 gene expression levels were selected.

MCMC oligogenic combined segregation and linkage analysis has been implemented in the program Loki [2]. These methods use linkage data on pedigrees and estimate the number, location, and effects of loci that contribute to a quantitative trait. These methods were designed for microsatellite marker maps, but have also been demonstrated to be practical with SNP markers [3]. The goal here was to examine the limits of these methods when applied to a sample the size of CEPH pedigrees, which is typical of many family studies.

## Methods

### Trait and marker selection

Traits were selected on several differing criteria, including heritability, linkage found with other methods, and random selection. Heritabilities were computed by Yu et al. [4] for all expression-level phenotypes provided. Eight phenotypes were selected with heritabilities ranging from near 0 to 0.87. In addition, Yu et al. [4] found linkage with Merlin nonparametric LOD scores >3 for several phenotypes, and three of these phenotypes were selected to see if those results could be replicated with these methods. In addition, one phenotype was selected at random. Finally, we simulated data from a random normal distribution to provide a "null" for comparison.

The meiotic map assembled by Sung et al. [5] guided the selection of SNP markers to include in our linkage analysis. Only markers present in the Rutgers map were used because that map was more complete. Furthermore, because the implementation of the methods used here does not address the issue of two markers with the same map position, when two markers had the same meiotic map position, we used only the first. A total of 1386 SNPs were selected with an average distance of 2.7 cM (0.01 cM minimum, 16.8 cM maximum). Although less than ideal, this marker set was judged sufficient for testing. There were a few Mendelian inconsistencies in the selected markers, which were resolved via the auto-correction feature in Loki.

### MCMC segregation and linkage analysis

To estimate the number, effects, and location of loci contributing to each phenotype, we applied the MCMC segregation and linkage analysis methods described by Heath [2]. These methods also estimate covariate effects, and the trait model is given by $y=\mu +X\beta +\sum_{i=1}^{k}Q_{i}\alpha_{i}+e$, where *μ *is the "reference" trait value, *X *is the incidence matrix for covariate effects, *β *is the vector of covariate effects, *Q*_*i *_is the incidence matrix for the effects of QTL *i*, *α*_*i *_is the vector of effects for QTL *i*, *e *is the normally distributed residual effect, and *k *is the number of QTL currently estimated (*k *≥ 0). The MCMC process samples *μ*, *β*, *α*_*i*_, *i*, and *e *as well as parameters such as unobserved marker genotypes. All of these parameters are sampled from the space of model values consistent with the data observed. Values are sampled proportional to their posterior probability. After a number of sampling iterations, the sampled values provide an estimate of the posterior probability distribution over the space of possible parameter configurations. Genome-wide analyses of each trait were run for 1,000,000 iterations with a LM sampler ratio of 0.2. In each analysis, all chromosomes were analyzed simultaneously. The raw (untransformed) traits were analyzed without covariates. Graphical analysis was used to assess MCMC mixing.

### Bayesian L-score

To evaluate evidence for linkage, we considered L-scores estimated over 1-cM wide bins along the chromosomes. An L-score is simply the posterior probability divided by the prior probability. In the absence of any data, the posterior probability should be equal to the prior probability. Thus, a L-score of 1 indicates that the data contains no information for or against linkage, while a L-score >1 indicates evidence for linkage, and an L-score <1 might be considered evidence against linkage.

## Results

The 12 traits and simulated null, selection criteria, heritabilities (*h*^2^), and a basic summary of the oligogenic segregation analysis are provided in Table 1. The maximum L-score on each chromosome for each trait and the location of the maximum L-scores >7.3 are in Tables 2 and 3. Also indicated are structural gene locations and locations of LOD scores >1.5 found by Yu et al. [4]. All 12 traits had L-scores suggestive of linkage: 11 had L-scores >30, and 3 had several >20. Only the randomly selected trait had no L-scores over 20, although it had an L-score of 17.57 on chromosome 3 and of 16.53 on chromosome 11. In another study, we found an empirical 95% chromosome-wide significance level at an L-score of 7.3, so we would normally follow up such linkage signals. The two traits (210910_s_at and 209480_at) with the largest *h*^2 ^had the largest percentage of genetic variance (%gv) in the oligogenic segregation analysis and also the largest L-scores we have encountered to date. In addition to these two, the three traits selected for LOD scores >3 also had L-scores >100, and in all five of these traits, the high L-score and LOD occur on the chromosome with the structural gene. In two other traits, the highest L-score is on the structural gene chromosome, but no others have a LOD >1.5 near the structural gene. In the five traits with no linkage to the structural gene, 27 L-scores >7.3 and 5 LOD scores >1.5 were found, but only two chromosomes had both. Overall, 42 L-scores >7.3 and 24 LOD scores >1.5 were found, including nine chromosomes with both. On these nine, the plausible interval for the L-score and the LOD-1 interval overlap on eight, and are ~35 cM apart on the ninth (chromosome 11 for 218264_at). In general, the L-score intervals are similar or smaller than the LOD intervals: on chromosome 1 for 204418_x_at, the two intervals are nearly identical, while on chromosome 7 for 210910_s_at, the L-score interval is ~6 cM in the middle of a ~17-cM LOD interval. No L-scores >7.3 were found for the simulated null. While some small *h*^2 ^values were selected, the smallest percentage of genetic variance for these traits from the oligogenic model was 24%. Consequently, it may not be surprising that evidence of linkage was found for all 12 phenotypes.

**Table 1:** Selection criteria, heritability (h^2^), and segregation results

			QTL			
			
Trait	Selection criteria	*h*^2^	No.^a^	Posterior probability of No.	pp ≥ 1^b^	% Genetic variance^c^
210910_s_at	Heritability	0.873	1	0.7510	>0.9999	0.808
209480_at	Heritability	0.858	2	0.5373	>0.9999	0.942
218435_at	Heritability	0.657	2	0.8459	>0.9999	0.678
220937_s_at	Heritability	0.501	2	0.8090	>0.9999	0.652
210797_s_at	Heritability	0.400	1	0.6213	0.8811	0.240
203395_s_at	Heritability	0.196	1	0.6598	0.8879	0.259
204234_s_at	Heritability	0.100	1	0.9324	>0.9999	0.489
212870_at	Heritability	0.000	2	0.9013	>0.9999	0.711
202546_at	Linkage	0.399	2	0.5659	0.9998	0.463
204418_x_at	Linkage	0.420	1	0.5602	>0.9999	0.584
33307_at	Linkage	0.383	2	0.5916	>0.9999	0.731
218264_at	Random	0.161	2	0.7519	>0.9999	0.578
Simulated	Simulation	0	0	0.7223	0.2777	0.020

^a^Number of trait loci with highest posterior probability

^b^The posterior probability of greater than one trait loci (pp ≥ 1)

^c^Percentage of variance due to genes in oligogenic segregation analysis

**Table 2:** Maximum L-scores and positions on each chromosome for expression phenotypes selected for LOD score >3, selected at random, and simulated null data

	Trait								
	
	202546_at (*h*^2 ^= 0.399; *p *= 0.1468)^a^		204418_x_at (*h*^2 ^= 0.420; *p *= 0.1439)		33307_at (*h*^2 ^= 0.383; *p *= 0.0002)		218264_at (*h*^2 ^= 0.161; *p *= 0.0063)		Simulated
Chr	Position (cM)^b^	L-score	Position (cM)	L-score	Position (cM)	L-score	Position (cM)	L-score	L-score

1	--	0.5	142.6^c, d^	477.7	--	3.6	--	1.2	0.8
2	115.5^c, d^	125.9	--	3.8	--	2.1	--	3.9	0.5
3	--	0.5	--^e^	1.0	25.5	19.1	39.5	17.6	0.5
4	--	0.5	--	1.9	--	3.2	--	2.2	0.5
5	--	0.5	--	1.1	--	2.5	--	4.7	0.8
6	--	0.5	--	0.8	--	1.4	--	6.6	0.4
7	--	1.7	--^f^	0.5	--	4.5	--	1.3	1.2
8	--	0.4	--	1.4	--	3.2	--	3.5	1.2
9	--	0.4	--^e^	0.5	--^f^	2.1	--	9.4	1.0
10	--	0.6	--	0.8	--	1.4	--^c^	1.1	0.9
11	--	0.4	--	0.9	--	1.4	46.5^e^	16.5	1.5
12	--	0.8	--	0.3	--	4.3	--	2.1	0.6
13	--	0.5	--	0.6	--	5.9	--	2.7	0.5
14	--	0.4	--^e^	1.8	--	1.6	--	2.1	0.6
15	--	0.4	--	0.7	--	2.0	--	1.9	0.4
16	--	0.5	--	0.4	--	0.7	--	1.1	1.1
17	--	0.5	--	0.6	--	0.7	--	2.7	0.7
18	--	0.4	--	0.3	--	1.2	--	1.6	0.5
19	--	0.4	--	1.3	--	0.5	5.1	9.7	0.5
20	--	0.4	--	0.4	--	0.7	--	0.9	0.4
21	--	0.5	--	0.8	--	2.2	--	6.0	0.7
22	--	0.5	--	0.6	61.5^c, d^	163.2	--	1.9	0.5

^a^Sharpiro-Wilk normal test *p*-value

^b^--, L-scores <7.3

^c^Chromosome with structural gene

^d^LOD > 3

^e^2 > LOD > 1.5

^f^3 > LOD > 2

**Table 3:** Maximum L-scores and positions^b ^(in cM) on each chromosome for expression levels selected on heritability

	210910_s_at (*h*^2 ^= 0.873; *p *= 0.0003)^a^		209480_at (*h*^2 ^= 0.858; *p *= 1.0475 × 10^-13^)		218435_at (*h*^2 ^= 0.657; *p *= 1.5142 × 10^-6^		220937_s_at (*h*^2 ^= 0.501; *p *= 0.0979)		210797_s_at (*h*^2 ^= 0.400; *p *= 0.3859)		203395_s_at (*h*^2 ^= 0.196; *p *= 0.0077)		204234_s_at (*h*^2 ^= 0.100; *p *= 0.6618)		212870_a (*h*^2 ^= 0; *p *= 0.0217)	
	
Chr	Pos^b^	L-scr	Pos	L-scr	Pos	L-scr	Pos	L-scr	Pos	L-scr	Pos	L-scr	Pos	L-scr	Pos	L-scr
1	--	0.9	--	3.2	--	2.1	--	3.1	--	2.5	--	1.5	78.6	15.8	--^c^	6.8
2	--^d^	0.3	174.5	8.2	--	0.9	150.5	20.4	--	4.4	152.5	12.7	33.5	15.8	--	5.7
3	--	0.7	--	0.9	68.5	13.7	--	1.7	--	4.5	--^e^	2.0	35.5	10.0	25.5	54.9
4	--	0.2	102.5	7.8	219.5	28.6	120.5	8.1	--	2.8	--	1.0	118.5	12.0	--	1.6
5	--	0.2	--	0.2	--	4.9	28.5	31.6	--	5.9	--	3.2	52.5	8.0	--	0.9
6	--	0.2	56.5^e, f^	1084.5	98.5	19.3	--	3.3	--	1.6	--	2.4	--	2.5	46.5	16.2
7	90.4^e, f^	1930.1	--	0.7	--	2.1	--	0.5	--	2.2	--	1.6	--	0.9	179.4	14.8
8	--	0.2	--	0.2	--	1.1	--	1.1	--	0.8	--	1.5	--^c^	5.9	--	2.8
9	--	0.3	--	0.2	--	4.9	--^e^	4.0	134.5^d^	16.2	--	5.5	--	2.5	--	1.3
10	--	0.3	--	2.9	--	4.3	--	0.5	--	1.6	148.5	39.1	--	5.4	--	0.7
11	--^d^	0.7	--	0.9	--^c^	5.1	26.5	9.9	--	3.1	--	1.5	--^e^	0.2	--	4.9
12	--	1.0	--	0.8	--	1.2	160.5	13.5	51.5^e^	39.4	--	6.9	--	0.9	--^e^	4.8
13	--	0.2	--^d^	0.3	41.5^e^	86.4	--	6.4	--	0.8	--	2.2	89.5	25.5	--	1.5
14	--	0.5	--^c^	0.2	--	0.3	--	7.0	--	4.4	--	3.0	--	2.2	92.5	7.4
15	--	0.2	--	0.5	--	3.3	--	2.1	--	2.8	--	3.6	--	1.7	--	0.7
16	--	0.2	--	0.8	--	3.8	45.0	20.1	65.0^c^	9.7	--	2.0	51.0	22.3	--	0.9
17	--	1.9	--	0.4	--	0.5	--	0.5	--	2.2	--	1.6	9.1	37.6	--	0.5
18	--	0.4	--	0.9	--^d^	3.9	5.4^d^	7.4	--	0.7	--^d^	3.2	--	1.5	--	5.3
19	--	0.2	--	0.5	--	3.5	--	1.9	--	1.5	--	2.8	--	1.8	--	1.6
20	--	0.2	--	0.2	--	0.4	32.7	9.4	--^d^	5.0	--	1.8	--	1.0	--	0.6
21	--	0.4	--	0.2	--	0.5	--	0.7	--	0.7	--	0.3	66.9	27.1	--	0.9
22	--	0.2	--	0.5	--	2.4	--	1.3	--	1.7	--	3.4	55.5	14.5	--	3.1

^a^Sharpiro-Wilk normal test *p*-value

^b^--, L-scores < 7.3

^c^3 > LOD > 2

^d^2 > LOD > 1.5

^e^Chromosome with structural gene

^f^LOD > 3

## Discussion

Our results suggest MCMC oligogenic segregation and linkage analysis may localize genes for traits with a variety of inheritance models. Some of these 12 traits appear to be essentially monogenic. For others, we found multiple linkage peaks. While the number of trait loci with the highest posterior probability was never >2, some traits produced >2 linkage peaks. The "extra" linkage peaks may not be false positives: it is possible that with this sample size, power is lacking to model more than two loci at once. As the sampler shifts over different parameters and numbers of loci, different loci may be modeled and localized, even if not all are in the model at once. If true, one would expect a larger sample size to have a larger mean for posterior probability on the number of loci. The possibility of false positives must always be considered. Whether to analyze raw or normalized data was considered here with some concern that non-normal distributions might contribute to false positives. A decision to focus resources on raw phenotypes was made, but we performed normality tests on all traits: the distributions of the two traits with the most L-scores >7.3 were found to be normal, while the two highest *h*^2 ^traits with very strong L-scores and LOD scores were found to be non-normal. These results are not conclusive, but suggest that data normality might not be the primary cause of false positives when using these methods. Also, the empirical 7.3 L-score value has not been validated in this data set. We expect an empirical cut-off value for this data would be similar, but computing this value takes many times longer than the analyses themselves. Setting this value too low would increase the false-positive rate.

While it was gratifying to find linkage for all 12 phenotypes, we were not able to determine the limits of these methods on a sample of this size exactly. In planning this study, it was expected that linkage would be found with an *h*^2 ^of 0.2, and possibly smaller. These results suggest *h*^2 ^may not always predict in which traits genes can be mapped. Traditional methods for computing *h*^2 ^might not adequately reflect genetic reality. It appears that the estimated percentage of genetic variation from the segregation part of the oligogenic analysis may be a better predictor. These results suggest that if percentage of genetic variation is >20%, we may be able to map a gene in a sample of this size. This percentage of variance can be quickly computed in an oligogenic segregation-only analysis. We were not able to determine whether we can map genes accounting for less variance in this sample or if a larger sample was needed. Also, the success seen here should be viewed with some caution when applied to general biological measures. A priori, one might expect gene expression levels to be under genetic control. In addition, these phenotypes were pre-screened, with over half the expression levels discarded before they were analyzed. It may be that *h*^2 ^is a generally a better predictor of finding linkage for arbitrary biological traits. However, these results suggest that there may be some traits in which low *h*^2 ^may not predict that no gene can be mapped.

## Conclusion

These results indicate that MCMC oligogenic segregation and linkage analysis may be useful in localizing genes for a variety of traits in a sample like that provided by the CEPH pedigrees. The percentage of trait variance estimated to be due to genes in an oligogenic segregation analysis may be a better predictor of the ability to map genes for that trait than a traditionally computed *h*^2^. It appears that we can localize genes accounting for ~20% of trait variance (and possibly less) in a sample of 14 families comprising 194 individuals. The LOD scores and the L-scores identified some regions in common and some individually. The L-score appears to do better than the nonparametric LOD by some measures (e.g., narrower intervals, linkages found at structural genes), but the individual results may indicate both types of analyses can be useful.

## Competing interests

The author(s) declare that they have no competing interests.

## References

[B1] Morley M, Molony CM, Weber TM, Devlin JL, Ewens KG, Spielman RS, Cheung VG (2004). Genetic analysis of genome-wide variation in human gene expression. Nature.

[B2] Heath SC (1997). Markov chain Monte Carlo segregation and linkage analysis for oligogenic models. Am J Hum Genet.

[B3] Daw EW, Heath SC, Lu Y (2005). Single-nucleotide polymorphism versus microsatellite markers in a combined linkage and segregation analysis of a quantitative trait. BMC Genet.

[B4] Yu R, DeHoff K, Amos C, Shete S (2007). Seeking gene relationships from gene expression data using support vector machines. BMC Proc.

[B5] Sung YJ, Di Y, Fu AQ, Rothstein JH, Sieh W, Tong L, Thompson EA, Wijsman EM (2007). Comparison of multipoint linkage analyses for quantitative traits in the CEPH data: parametric LOD scores, variance components LOD scores, and Bayes factors. BMC Proc.

